# Optical properties of folic acid in phosphate buffer solutions: the influence of pH and UV irradiation on the UV-VIS absorption spectra and photoluminescence

**DOI:** 10.1038/s41598-019-50721-z

**Published:** 2019-10-03

**Authors:** Mihaela Baibarac, Ion Smaranda, Andreea Nila, Constantin Serbschi

**Affiliations:** 1 National Institute of Materials Physics, Laboratory of Optical Processes in Nanostructured Materials, Atomistilor street 405A, Magurele, RO-77125 Romania; 2 Bioelectronic SRL, Cercelus street, no.54, Ploiesti, Romania

**Keywords:** Photobiology, Surfaces, interfaces and thin films

## Abstract

Using UV-VIS absorption spectroscopy, photoluminescence (PL) and photoluminescence excitation (PLE), the photodegradation reactions of folic acid (FA) in phosphate buffer (PB) solutions were studied. Regardless of the PB solution’s pH, the UV-VIS spectra showed a gradual decrease in absorbance at 284 nm simultaneous with an increase in the absorbance of another band in the spectral range of 320–380 nm, which was downshifted under UV irradiation. The relative intensity of the FA PL band, situated in the spectral range 375–600 nm, was dependent on the pH of the PB solution. The FA PL intensity increased as increasing UV irradiation time up to 281 min. in PB solutions with pH values of 6.4 and 5.4. Under an emission wavelength of 500 nm, the position of the FA PLE spectrum changed as the PB solution pH varied from 7 to 5.4 and the irradiation time increased to 317 min. These changes were correlated with the formation of two photodegradation products, namely, pterine-6-carboxylic acid and p-amino-benzoyl-L-glutamic acid. According to UV-VIS spectroscopy and PL and PLE studies, the presence of various excipients in commercial pharmaceutical tablets does not affect the photodegradation of FA in PB solutions. Using IR spectroscopy, new evidences for the formation of the two photodegradation products of FA in PB solutions are shown.

## Introduction

Research on the physical and chemical properties of folic acid (FA) and its therapeutic effects dates to 1944^1,2^. At present, this drug is administered to pregnant women with FA deficiency to avoid neural tube defects in newborn babies and various treatments for cardiovascular diseases, Alzheimer’s disease, cancer, anemia and so on^3,4^. The amount of FA administered to each patient is dependent on their health history and age. The development of various analytical methods for the determination of FA has been reported, including high-performance liquid chromatography (HPLC)^5^, electrochemical techniques such as cyclic voltammetry^6^, UV-VIS spectroscopy^7^, fluorescence^8^, and surface enhanced Raman scattering^9^. A large number of platforms for FA determination have been developed, including i) carbon paste electrodes modified with gold nanoparticles^10^, (ii) hybrid structures based on reduced graphene oxide and silver nanoparticles^11^, (iii) single-wall carbon nanotubes functionalized with ionic liquids and multiwall carbon nanotube paste electrodes^12,13^; (iv) graphene/ZnO nanowire arrays^14^; (v) magnetic nanocomposites based on Fe_3_O_4_ – ZnS: Mn^2+^/SiO_2_-NH_2_^15^, MoS_2_/reduced graphene oxide^16^ and (vi) phosphomolybdic acid-polypyrrole/graphene composites^17^. Various strategies have been applied for the simultaneous determination of FA and the following chemical compounds of interest in the medical field: (i) epinephrine and uric acid^18^, (ii) tryptophan, L-cysteine and acetaminophen^19^ and (iii) ascorbic acid, dopamine and uric acid^20^.

Despite this progress, little data about FA photodegradation have been reported^21–24^. This topic was first discussed in 1999, when a study using HPLC identified two products of FA photolysis: pterine-6-carboxylic acid and p-amino-benzoyl-L-glutamic acid^21^. A reaction mechanism proposed for the FA photodegradation in acid and alkaline media was reported in 2003^22^. In order to understood this process, the identification of the photoproducts resulted under UV photodegradation when FA was dissolved in phosphate buffer (PB) solutions having pH equal with 7 was studied by UV-VIS spectroscopy, photoluminescence (PL) and photoluminescence excitation (PLE)^23,24^. Taking into account the presence of FA in various media like urine, human blood serum, milk, fruit juice and so on, a part from these having the pH values under 7, in the present work the FA photodegradation in PB solutions having pH equal with 6.4 and 5.4 will be assessed by reporting at the reference samples having pH equal with 7 using UV-VIS absorption spectroscopy, PL and PLE.

The photodegradation of FA in PB having pH equal to 6.4 and 5.4 was studied by the UV-VIS spectroscopy under the UV irradiation. Additional information about this photodegradation process of the FA samples in PB having pH equal to 6.4 and 5.4 will be shown by PLE and PL studies. In this order using the emission wavelength equal to 500 nm, the evolution of PLE spectra of FA samples in PB having pH equal to 6.4 and 5.4 in the UV spectral range, namely between 275 and 480 nm, will be reported. Using the excitation wavelength equal to 335 nm, the evolution of PL spectra of FA samples in PB having pH equal to 6.4 and 5.4 in the spectral range between 350 and 650 nm will be reported, too.

At present, in pharmaceutical products the FA tablets contain additional chemical compounds, including lactose monohydrate, magnesium stearate, butylhydroxyanisole, polysorbate 80, iron oxide, microcrystalline cellulose, and titanium dioxide. We demonstrated that UV-VIS spectroscopy and PL can be used to investigate FA photodegradation in the presence of various excipients.

Using IR spectroscopy, new evidences for the two photodegradation products of FA are shown.

The used protocol to study photodegradation of FA in this work was that published by Welankiwar *et al*.^25^.

## Results and Discussion

**Figure 1:**
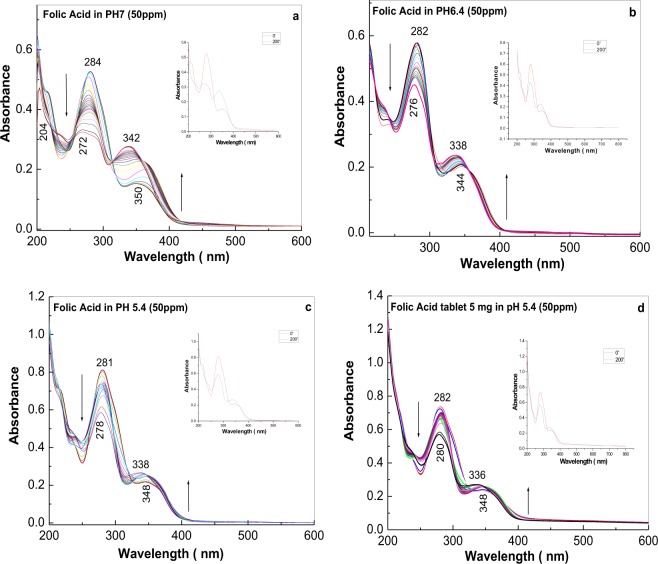
UV-VIS absorption spectra of folic acid in phosphate buffer having pH equal with 7 (**a**), 6.4 (**b**) and 5.4 (**c**) recorded after UV irradiation times of 0, 2, 4, 6, 8, 10, 12, 14, 16, 18, 20, 22, 24, 26, 28, 30, 32, 34, 36, 38, 40, 50, 70, 100, 160 and 200 min. (**d**) shows the UV-VIS absorption spectra of an folic acid tablets in phosphate buffer solution having pH equal with 6.4. All samples included 50 ppm folic acid. All figures show inserts with UV-VIS spectra before and after 200 min. of the UV irradiation in order to better highlight the isosbestic points.

Figure 1 shows the UV-VIS absorption spectra of FA in PB solutions having pH equal with 7, 6.4 and 5.4 and their evolution after UV irradiation at a wavelength of 253 nm for 200 min. All FA solutions used for the studies shown in Fig. 1 were at a concentration of 50 ppm. In the initial state, regardless of the PB solution pH, two absorption bands appeared in the spectral ranges 200–300 and 300–400 nm, which were assigned to the π-π* and n-π* electronic transitions, respectively, of the pterin (Pt) and p-amino benzoyl acid (PABA) moieties of FA^26^. Without UV irradiation, decreasing the pH of the PB solution from 7 to 6.4/5.4 induced a downshift of the two absorption bands from 284 and 350 nm to 282/281 and 344/348 nm, respectively. In addition, Fig. 1 shows that as the UV irradiation time increased, the absorbance of the band assigned to the π-π* electronic transition gradually decreased, while the absorbance of the band assigned to the n-π* electronic transition progressively increased in FA solutions in PB at pH 7, 6.4 and 5.4. This behavior induces the appearance of an isosbestic point, which in the case of FA solutions in PB having pH values of 7, 6.4 and 5.4, is situated at wavelengths 316, 312 and 310 nm, respectively. This observation indicates the presence of additional compounds, besides FA, were formed by a photochemical reaction that occurs in aerobic conditions. The mechanism for such a photochemical reaction was proposed by Akhtar *et al*.^22^, who suggested, based on HPLC studies, that two photodegradation products, namely, pterine-6-carboxylic acid (PCA) and p-amino-benzoyl-L-glutamic acid (PABGA), are formed. The aerobic total reaction which results in the formation of pterine-6-carboxylic acid and p-amino-benzoyl-L-glutamic acid can be written as in Schema 1S. The chemical mechanism of the reaction shown in Scheme 1S considering the FA molecular structure was discussed by M. J. Akhtar *et al*.^22^.

The photochemical reaction rate of FA can be described using the following equation: ln C_t_ = ln C_0_ − kt, where C_t_, C_0_, k and t correspond to the FA concentration at the various irradiation times and before the irradiation process, reaction rate and the illumination time, respectively^27^. The plotting of lnC versus t highlights a linear dependence, which shows a slope equal to -k corresponding the pseudo-first order rate constant^27^. Considering the Lambert-Beer law and the UV-VIS spectra shown in Fig. 1, in the case of the FA samples in PB solutions having pH equal to 7, 6.4 and 5.4, the values for k of 0.0163, 0.0022 and 0.0019 min^−1^, respectively, are reported.

With UV irradiation, a downshift of the two FA absorption bands in PB solutions was observed (Fig. 1). At pH 7, the bands at 284 and 350 nm shifted to 272 and 342 nm, respectively, while at pH 6.4, the bands shifted from 282 and 344 nm to 276 and 338 nm, respectively. At pH 5.4, the bands shifted from 281 and 348 nm to 278 and 338 nm, respectively. Regardless of the PB solution pH, an increase in the number of photons absorbed is reported. As the irradiation time increases, regardless of the pH of the PB solution, the number of photons absorbed increases, too. Thus, for FA in the PB solution having pH equal to: (i) 7, after 2, 50, 100 and 200 minutes of UV irradiation, the number of photons absorbed is estimated to be equal to 6,008 × 10^22^, 1,459 × 10^23^, 2.87 × 10^23^ and 5,751 × 10^24^, respectively; (ii) 6.4, after 2, 50, 100 and 200 minutes of UV irradiation, the number of photons absorbed is calculated to be 5.963 × 10^22^, 1.491 × 10^23^, 2.981 × 10^23^ and 5.836 × 10^24^, respectively; and (iii) 5.4, after 2, 50, 100 and 200 minutes of UV irradiation, the number of photons absorbed is equal to 5,941 × 10^22^, 1,501 × 10^23^, 2,961 × 10^23^ and 5,878 × 10^24^, respectively. A similar pattern was observed in the case of commercial tablets with 5 mg FA. This observation indicates that the FA photochemical reaction in aerobic conditions is not inhibited by the presence of various excipients.

Considering the experimental UV-VIS spectra of FA in PB solutions having pH equal to 7, 6.4 and 5.4 (Fig. 1a–c), using DATAN5.1 package, the concentration of the protolytic species depending on the solution pH and acidity constants, were assessed. According to Fig. 1S, in the case of the solutions with pH equal to 7, 6.4 and 5.4, the ratio between the molar concentrations of protolytic species of FA at each pH resulted in pKa values equal to 6.79 and 6.22 in the case of samples before and after UV irradiation, respectively.

**Figure 2:**
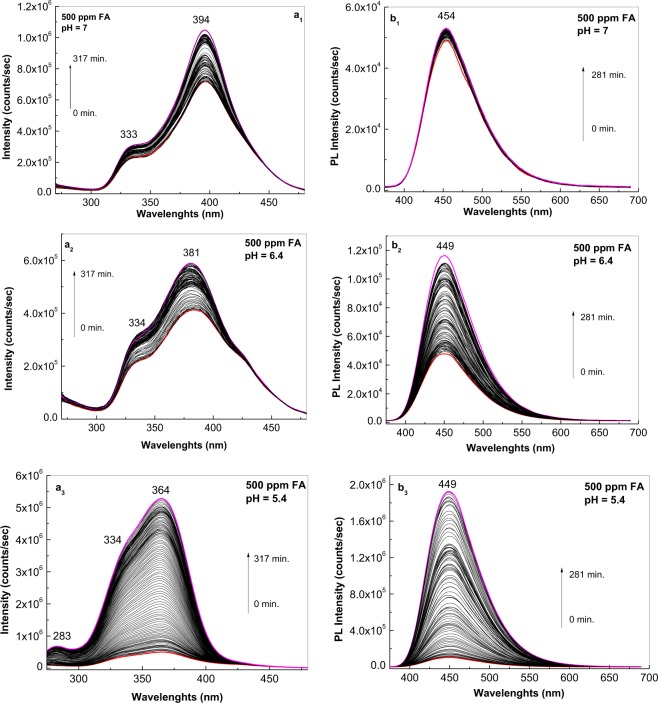
Photoluminescence excitation (**a**, the emission wavelength (λ_em_) is equal to 500 nm) and photoluminescence (**b**, the excitation wavelength (λ_exc_) is equal to 355 nm) spectra of a folic acid solution at 500 ppm in phosphate buffer at pH equal to 7 (a_1_, b_1_), 6.4 (a_2_, b_2_) and 5.4 (a_3_, b_3_), in the initial state (red curves) and after irradiation for 317 min and 280 min. (magenta curves), respectively. Black curves correspond to the intermediate photoluminescence excitation and photoluminescence spectra of the folic acid solution above phosphate buffers collected each at 141 and 125 seconds, respectively.

Additional information concerning the photochemical reactions of 500 ppm FA in PB solution having pH values equal with 7, 6.4 and 5.4 are shown in Fig. 2. A careful analysis of the PLE spectra of FA in PB having pH equal to 7, 6.4 and 5.4 before the irradiation process indicated: (i) the position of the PLE band maximum ranged from 394 nm to 381 nm and 364 nm; (ii) a change in the ratio between the intensity of the PLE bands present in the spectral ranges 375–500 and 300–375 nm from 3.38 to 1.88 and 1.41, respectively, was observed and (iii) the ratio between the intensity of the PLE bands peaked at 276 and 364 nm is equal with 5.36 in the case of the FA in PB having pH equal to 5.4. Depending on the solution pH, as the irradiation time was increased to 317 min., the following changes were observed (Fig. 2): (i) an increase in the relative intensity of all PLE spectra, the most significant increase (approximately 10.5-fold) being observed at pH equal with 5.4 (Fig. 2a_3_); and (ii) an up-shift of the PLE band of low intensity from 276 nm to 283 nm (Fig. 2a_3_). A consequence of this fact is that after 317 min. of irradiation, the ratio between the relative intensity of PLE bands at 364 and 283 nm was equal to 7.49 (Fig. 2a_3_).

**Figure 3:**
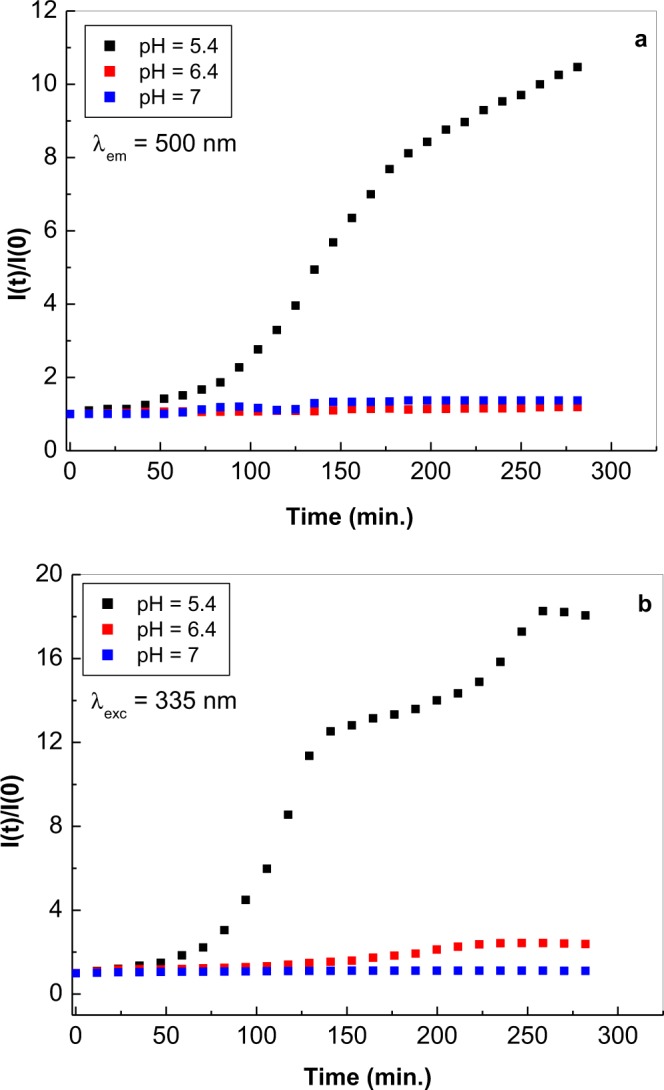
The dependence of the photoluminescence excitation (**a**) and photoluminescence (**b**) spectra intensities of the folic acid samples, having the concentration equal to 500 ppm in phosphate buffer at pH equal to 7 (blue squares), 6.4 (red squares) and 5.4 (black squares), with the irradiation time. Photoluminescence excitation and photoluminescence spectra were recorded at wavelengths equal to 500 and 335 nm, respectively.

As shown in Fig. 3, in FA solutions having pH equal with 5.4, an approximately 18-fold increase in the relative intensity of the PL band occurs. This value is superior at those reported in the case of FA solutions having pH equal to 6.7 and 7, for which an increase of 2.39 and 1.11-fold is reported (Fig. 3). An increase of 10.47, 1.37 and 1.2-fold was reported to take place in the case of PLE spectra of FA solutions in PB with pH equal to 5.4, 6.7 and 7, respectively (Fig. 3). In the case of the FA powder, under UV irradiation an increase of 1.37 and 1.27-fold was reported in the case of PLE and PL spectra recorded in the presence of the oxygen from air (Fig. 2S).

**Figure 4:**
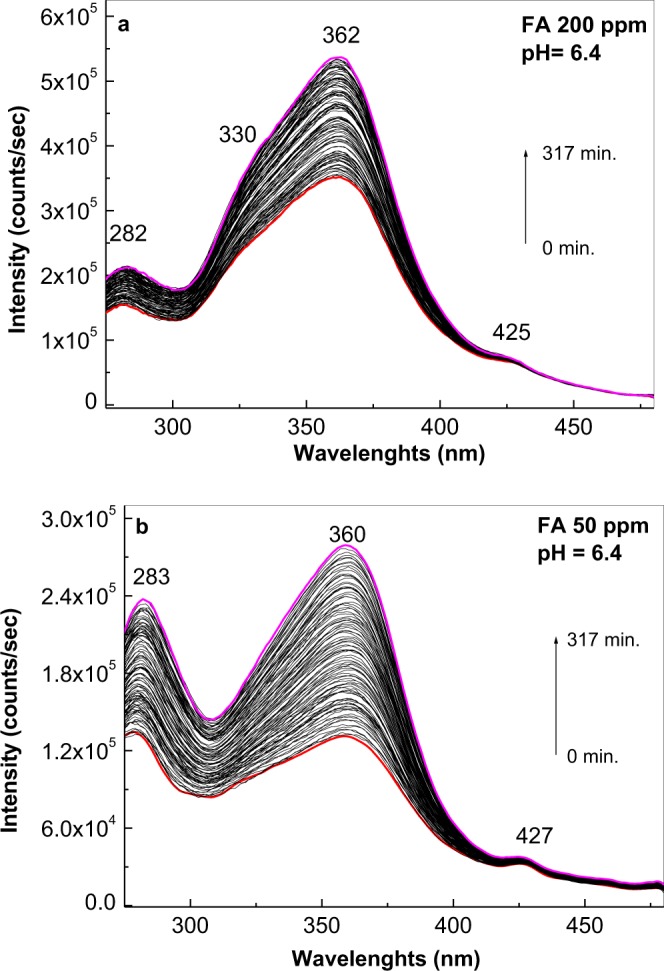
Photoluminescence excitation (the emission wavelength is equal to 500 nm) spectra of the folic acid samples at 200 (**a**) and 50 (**b**) ppm in phosphate buffer having pH equal with 6.4 in the initial state (red curves) and after irradiation for 317 min. (magenta curves). Black curves correspond to the intermediate photoluminescence excitation spectra of the folic acid solution in above phosphate buffers collected each at 141 seconds.

**Figure 5:**
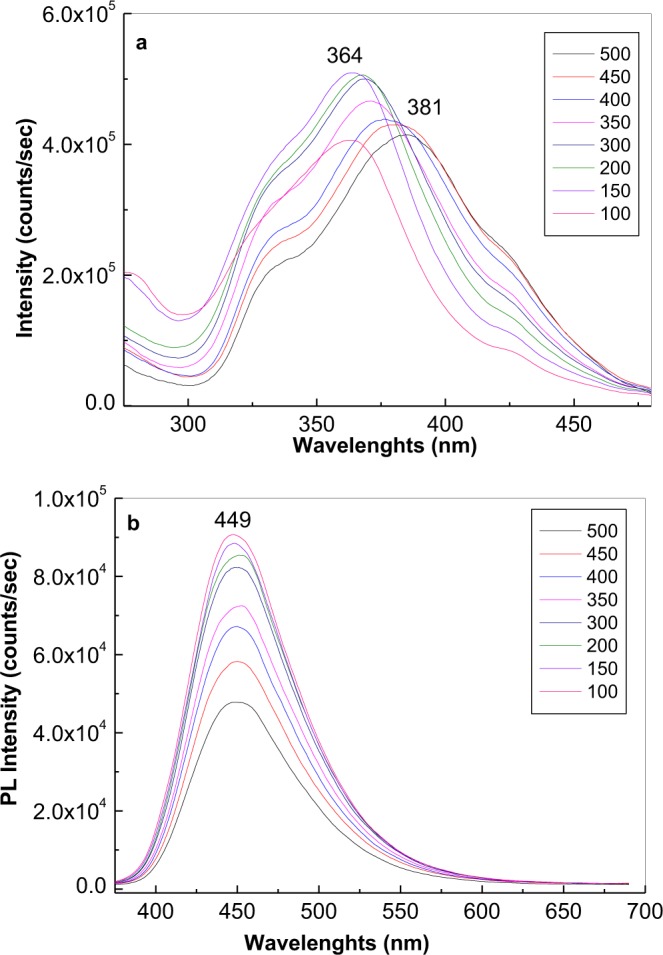
Photoluminescence excitation (**a**, the emission wavelength is equal to 500 nm) and photoluminescence (**b**, the excitation wavelength is equal to 355 nm) spectra of folic acid at 500, 450, 400, 350, 300, 200, 150 and 100 ppm in phosphate buffer having pH equal to 6.4 after irradiation time of 317 and 280 minutes, respectively.

The PLE band at 283 nm appeared with (i) less concentrated solutions of FA in PB at pH of 5.4 (Fig. 2a_3_), and (ii) 50–200 ppm solutions of FA in PB at pH of 6.4 (Fig. 4). In this last case, after 317 min. of irradiation, the ratio between the relative intensities of PLE bands at 362–360 and 283–282 nm were equal to 2.35 and 1.09, respectively. An upshift of the PLE band from 381 nm (3.25 eV) to 364 nm (3.41 eV) was observed as the FA concentration was decreased from 500 to 100 ppm at pH 6.4 (Fig. 5a). These changes origin in the acid-base interactions between the two constituents of the studied systems^28^.

For PL spectra of FA in PB solutions having pH equal with 7, 6.4 and 5.4 (Fig. 2), before UV irradiation, the emission band intensity varied from 48.280 counts/sec to 48.044 and 124.350 counts/sec, the maxima being at 454 nm, 449 nm and 449 nm, respectively. According to Fig. 5, a decrease in the FA concentration from 500 to 100 ppm in PB with pH of 6.4 induces an increase in the relative intensity of the PL band at 449 nm. After 281 min. of UV irradiation of FA in PB having pH equal with 7, 6.4 and 5.4, the intensity of the emission bands reached 54.560, 117.080 and 1.935.270 counts, respectively.

**Figure 6:**
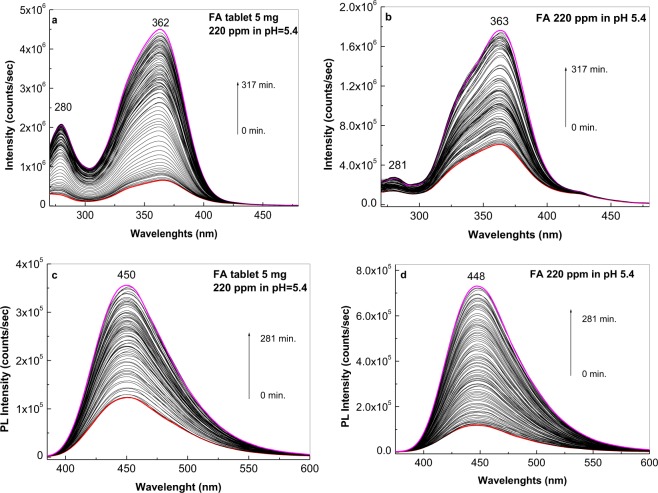
Photoluminescence excitation spectra of folic acid solutions (the emission wavelength is equal to 500 nm) at 220 ppm in phosphate buffer at pH 6.4 prepared using 5 mg commercial pharmaceutical tablets (**a**) and powder purchased form Sigma-Aldrich (**b**) after irradiation times of 317 minutes. Photoluminescence spectra of the folic acid solutions (λ_exc_ = 355 nm) at 220 ppm in phosphate buffer pH 6.4 prepared using 5 mg commercial pharmaceutical tablets (**c**) and powder purchased form Sigma-Aldrich (**d**), after irradiation times of 280 minutes. The photoluminescence excitation and photoluminescence spectra of the folic acid samples before of irradiation correspond to red curves. Magenta curves correspond to the photoluminescence excitation and photoluminescence spectra of the folic acid samples after an irradiation time equal to 317 and 280 min., respectively. Black curves correspond to the intermediate photoluminescence excitation and photoluminescence spectra of the folic acid solution in above phosphate buffers collected each at 141 and 125 seconds, respectively.

Figure 6 shows the PLE and PL spectra of 220 ppm FA in PB at pH 5.4 with and without excipients. Although the two solutions of FA had the same concentration, both PLE and PL spectra showed different intensities in the initial state and after irradiation. In the PLE spectra of FA solutions prepared from FA powder purchased from Sigma-Aldrich and commercial pharmaceutical tablets, the intensities of the dominant bands at 363 nm were 624.746 and 683.764 counts/sec. After 317 min. of irradiation, the PLE spectra intensities of the FA solutions prepared from powder purchased from Aldrich Sigma and commercial pharmaceutical tablets were 1.777.867 and 4.629.796 counts/sec, respectively. Detailed analysis of the PL spectra of FA solutions prepared from the powder purchased from Aldrich Sigma and commercial pharmaceutical tablet indicated that in the initial state, the emission band intensities at 450 nm were 84.510 and 120.410 counts/sec, respectively, after 280 min. irradiation, the intensities of the emission bands at 450–448 nm were 718.230 and 357.900 counts/sec, respectively.

**Figure 7:**
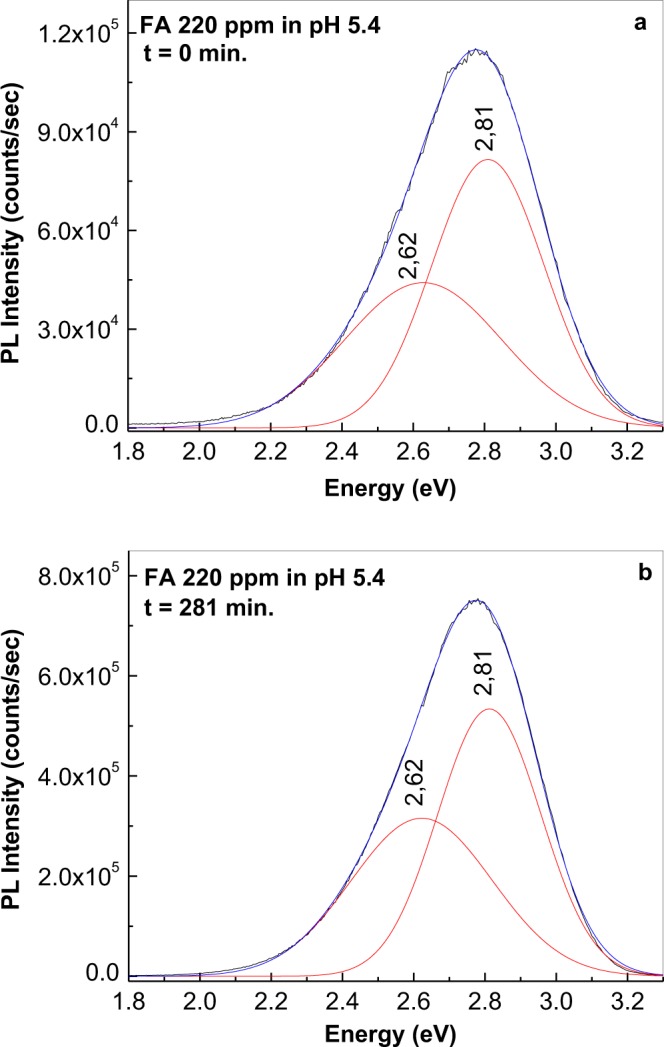
Spectral composition of photoluminescence spectra of folic acid in phosphate buffer at pH 5.4 in the initial state (**a**) and after 281 minutes of UV irradiation (**b**).

Figure 7 shows the deconvolutions of the PL spectra of an FA sample at 220 ppm in PB having pH equal with 5.4 before and after UV irradiation at 281 nm. Two emission bands with peaks at 2.62 and 2.81 eV were observed. The frequency separation between the emission bands at 2.62 and 2.81 eV was 0.19 eV, which corresponds to the Raman line of FA with maximum at 1536 cm^−1^, which was assigned to the (NH_2_) + stretching (C=N) + rocking ρ(CH) + (CH_2_) (pterine) vibrational mode^29^. Based on these data, we suppose that the increase in the intensity of the two emission bands at 2.62 and 2.81 eV indicates that FA photodegradation induces changes associated with the CH_2_ group that connects the pterine (Pt) moiety with p-amino-benzoyl amide (PAB). The fact that a similar behaviour was observed with FA from commercial pharmaceutical tablets under UV light shows that these photochemical reactions are not disrupted by the presence of excipients.

The increasing in the relative intensity of the FA PLE and PL spectra more pronounced in the presence of the PB solution having pH equal to 5.4 than at pH 6.4 or 7, origin the protolytic reactions of the two photodegradation products, when the complete transformation of the NH_2_ group, which plays the role of neutral base, into a NH_3_^+^X^−^ group (X^−^ = H_2_PO_4_^−^), that corresponds to a cationic acid, takes place. Scheme 2S shows the protolithic reaction of the two photodegradation products.

**Figure 8:**
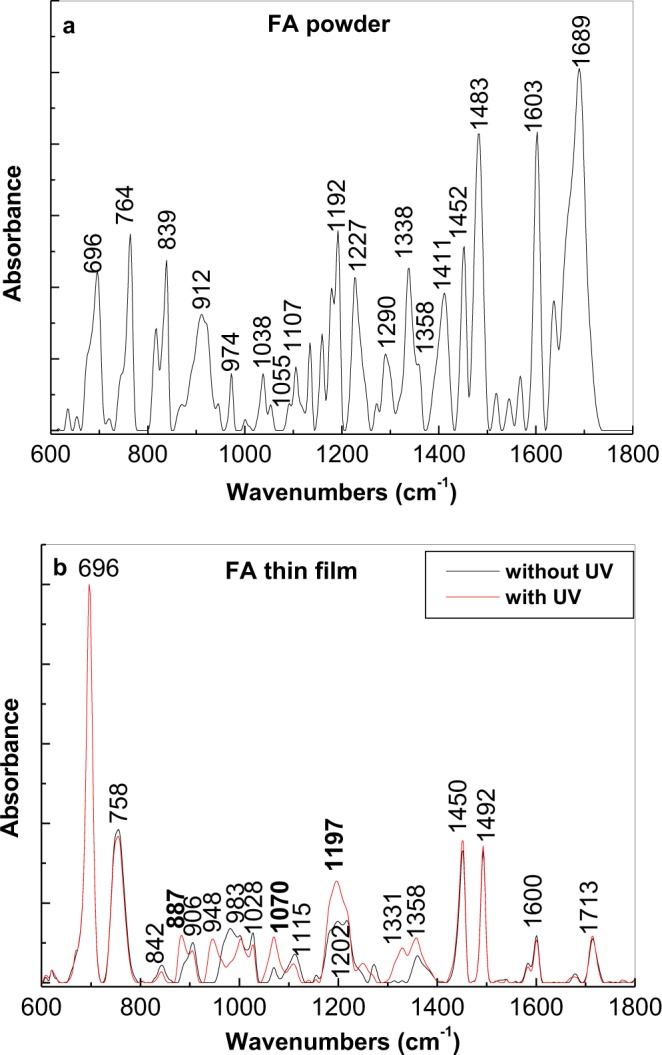
IR spectra of the folic acid in powder state (**a**) and as a thin film (**b**) made from 500 ppm folic acid solutions in phosphate buffer at pH equal with 5.4 before (black curve) and after a UV irradiation time of 200 minutes (red curve).

Additional information concerning the reaction products of FA photodegradation was uncovered by IR spectroscopy. Figure 8 shows the IR spectra of FA in the powder state and as thin film deposited onto a rough Au support, prepared according to a protocol described previously^30^. The IR absorption bands of FA in a powder state are at 696, 764, 839, 912, 974, 1038–1055, 1107, 1192, 1227, 1290, 1338, 1411, 1452, 1483, 1603 and 1689 cm^−1^. These IR bands were assigned according to their vibrational modes to the three moieties of FA, namely, Pt, PAB and glutamic acid (GA) (Tables 1S–3S).

**Figure 9:**
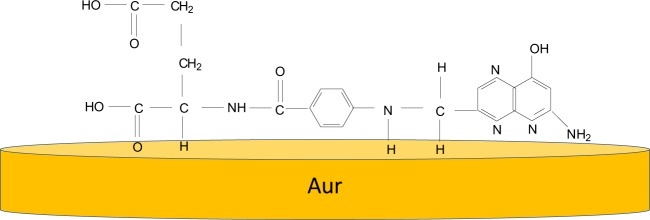
Adsorption of folic acid molecules onto the gold support.

Significant changes were noted when FA was deposited as thin film onto a rough Au support. As shown in Fig. 8, the main differences between the IR spectra of FA in the powder and thin film state were (i) a change in the ratio between absorbance of IR bands from 600–800 and 800–1800 cm^−1^ in favour of the former; (ii) IR bands in the range of 710–800 cm^−1^, assigned to the C-H out-of-plane deformation vibrational mode of the alkyl group in GA, was downshifted from 764 to 758 cm^−1^; (iii) the ratios between IR bands with peaks at 696 and 764/758 cm^−1^ were 0.79 and 2.72 with FA is in the powder and thin film states, respectively; iv) the IR bands at 912 and 1038 cm^−1^ were downshifted to 906 and 1028 cm^−1^, respectively; v) disappearance of the IR band at 1338 cm^−1^ when FA was deposited as a thin film; vi) an upshift of the IR band localized in spectral ranges 1460–1500 and 1650–1750 cm^−1^ from 1483 and 1689 cm^−1^ to 1492 and 1713 cm^−1^, respectively; and vii) the ratio between the absorbance of IR bands 1452/1450 and 1483/1492 cm^−1^ changed from 0.61 to 1.03, when FA was in the powder and thin film state, respectively. All these changes suggest a preferential orientation of the Pt, PAB and GA entities of FA onto the rough Au support, as shown in Fig. 9.

To determine the influence of UV light on FA in PB having pH equal with 5.4, we analysed the IR spectra of thin film of FA deposited onto an Au support before and after 281 min. of UV irradiation. To explain all the changes shown in Fig. 8, Tables 2S and 3S show the calculated vibrational modes of pterine-6-carboxylic acid (PCA) and p-amino-benzoyl-L-glutamic acid (PABGA). According to the black and red curves in Fig. 8, the main differences induced by UV light on the IR spectrum of FA were (i) an increase in the relative absorbance of the IR bands at 887 and 1070 cm^−1^ assigned to the C-H vibrational modes in the benzene ring and the NH scissor in the amine group of PABGA; (ii) the appearance of IR bands at 948 and 1331 cm^−1^ attributed to the C-C bending vibrational modes in alkyl and benzene ring and C=O stretching in COOH groups and C-H in the benzene ring and NH in amine group of PABGA; and (iii) an increase in the absorbance of the IR band at 1175–1225 cm^−1^, which was assigned to the O-H vibrational mode in the COOH group and the O-H in substituted aromatic ring of PCA. The increase in the absorbance of the IR bands at 948, 1070, 1331 and 1197 cm^−1^ indicates an increase in the mass of the amine and carboxyl groups in the analysed sample, which is a consequence of the photodegradation reaction of FA, as shown in Schema 1S.

## Conclusions

This work reports new data based on UV-VIS absorption spectroscopy and photoluminescence concerning the photochemical reactions of FA in PB solutions. New experimental evidences, concerning the photodegradation products resulted under UV irradiation of FA in PB solutions, are shown by IR spectroscopy for the first time. Our results allow to conclude that:

(i) under UV irradiation of FA in PB solutions at pH 7, 6.4 and 5.4 in the presence of oxygen from the air, a photochemical reaction takes place;
(ii) the appearance of an isosbestic point in UV-VIS spectra of FA in PB solutions at pH 7, 6.4 and 5.4 under UV irradiation indicates the formation of new chemical compounds as a consequence of the photochemical reaction in aerobic conditions;
(iii) the increase in the absorbance of the IR bands at 948, 1070, 1331 and 1197 cm^−1^, as UV irradiation time increases of FA in PB solution having pH equal to 5.4, are new evidences for the formation of the two photodegradation products of FA, i.e. pterine-6-carboxylic acid and p-amino-benzoyl-L-glutamic acid and
(iv) PLE and PL spectra are valuable tools in monitoring photochemical reactions of FA; the pronounced changes in PLE and PL spectra of FA in PB solution having pH equal to 5.4 were assigned to the protolithic reactions of pterine-6-carboxylic acid and p-amino-benzoyl-L-glutamic acid.

The results indicate that the handling of FA samples for medical analysis and in FA electrochemical/optical detection must be done in the absence of UV light.

## Methods

Chemical compounds, i.e., folic acid, sodium dihydrogen phosphate and sodium hydrogen phosphate, were purchased from Sigma-Aldrich. PB solutions at pH 7, 6.4 and 5.4 were prepared by mixing stock standard solutions of NaH_2_PO_4_ and Na_2_HPO_4_ × 12H_2_O.

FA tablets with 5 mg active compound were purchased from a local drugstore. The FA tablets were ground and dispersed in PB solutions, sonicated for 30 min. and then filtered to obtain a clear solution

UV-VIS spectra of FA in PB solutions were recorded using a Lambda 950 model UV-VIS-NIR spectrophotometer (Perkin Elmer). The FA photodegradation in PB solutions having pH equal to 7, 6.4 and 5.4 was done under an UV irradiation using a 350 W mercury-vapor lamp endowed with an optical filter UG5 which allow to select the 200–380 nm spectral range, where there is the most intense Hg spectral lines at 253 nm.

PL and PLE spectra of FA in PB solutions were recorded with right angle geometry at room temperature using a Fluorolog-3, FL3–2.2.1 spectrophotometer with a 450 W Xe lamp (Horiba Jobin Yvon). All PL and PLE spectra were recorded using excitation and emission slits equal to 1.5.

IR spectra of FA before and after UV irradiation were recorded using a Vertex 80 FTIR spectrophotometer (Bruker). For the IR absorption spectra of FA, assignment of only the IR bands situated in the spectral range 1450–3500 cm^−1^ has been reported. To better understand the changes to FA induced by UV irradiation, we calculated the IR vibrational modes of FA and its photolysis products. Optimization calculations of vibrational modes were performed using the SIESTA simulation package^31^. For the atomic structure relaxation, the correlation and exchange function was used for the local density approximation (LDA) proposed by Ceperley and Alder (CA)^28^; the molecular geometries were relaxed until the minimum energy on each atom was less than 0.1 meV/A. This equilibrium configuration is essential for obtaining a phononic database that matches the molecular structure. The set of k-points in the Brillouin zone (set as 4 × 4 × 1), as well as the mesh cutoff (set to 500 Ry) adopted in real space, were determined by convergence tests obtained after solving the equation with a single electron, taking into account the total energy fluctuation in terms of atom displacement. After obtaining the relaxed molecular structures, the vibrational modes were calculated based on a dynamic matrix with a finite differential calculus of the forces using the VIBRA^32^ package, which is part of the SIESTA package distribution.

## Supplementary information

Supplementary information
