# Supplementary material for: Optical properties of folic acid in phosphate buffer solutions: the influence of pH and UV irradiation on the UV-VIS absorption spectra and photoluminescence
Source: Sci Rep. 2019 Oct 3;9:14278. doi: 10.1038/s41598-019-50721-z (PMC6776545; doi:10.1038/s41598-019-50721-z)
Supplement: Supplementary file 1 — Supplementary information [file 41598_2019_50721_MOESM1_ESM.pdf]

Supplementary Information

Optical properties of folic acid in phosphate buffer solutions: the influence of pH and UV irradiation on the UV-VIS absorption spectra and photoluminescence

Mihaela Baibarac[1]*, Ion Smaranda[1], Andreea Nila[1] and Constantin Serbschi[2]

[1]National Institute of Materials Physics, Laboratory of Optical Processes in Nanostructured Materials Physics, Atomistilor street 405A, RO-77125, Magurele, Romania.

[2]Bioelectronic SRL, Cercelus street, no.54, Ploiesti, Romania

Corresponding author: Dr. M. Baibarac

Fax : + 40 21 3690177

Tel: + 40 21 3690170

E-mail: barac@infim.ro

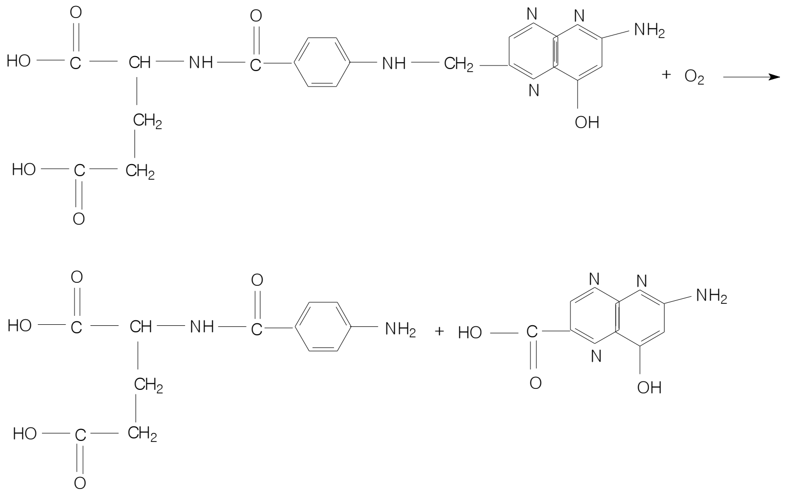

Scheme 1S. The photochemical reaction of FA in the presence of oxygen from air

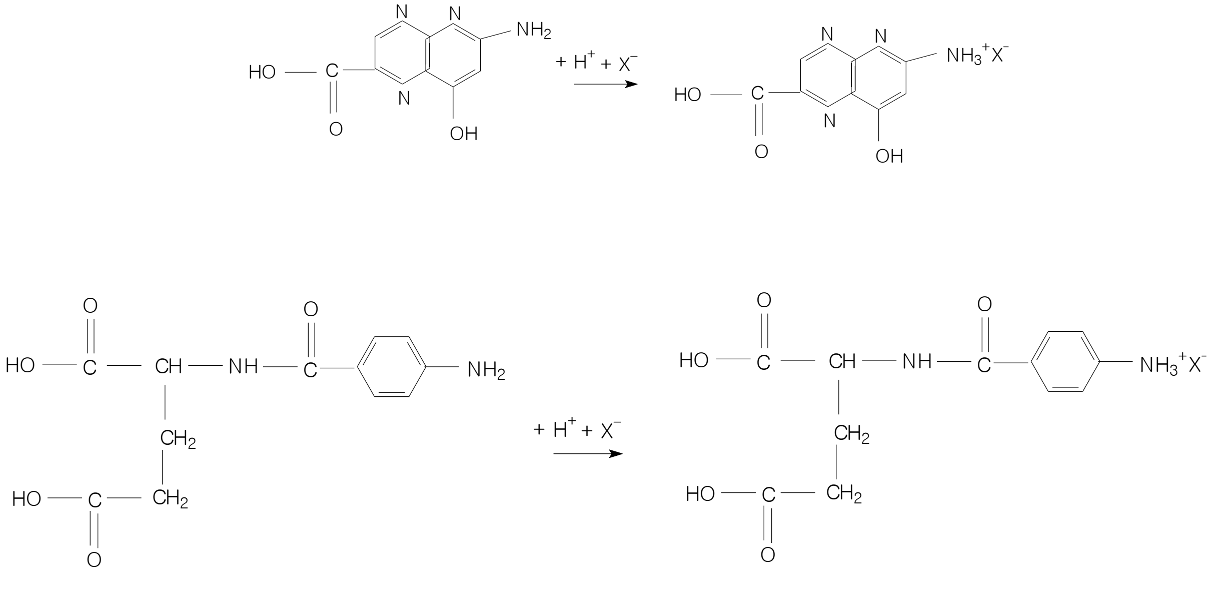

Scheme 2S. The protolithic reactions of pterine-6-carboxylic acid and p-amino-benzoyl-L-glutamic acid

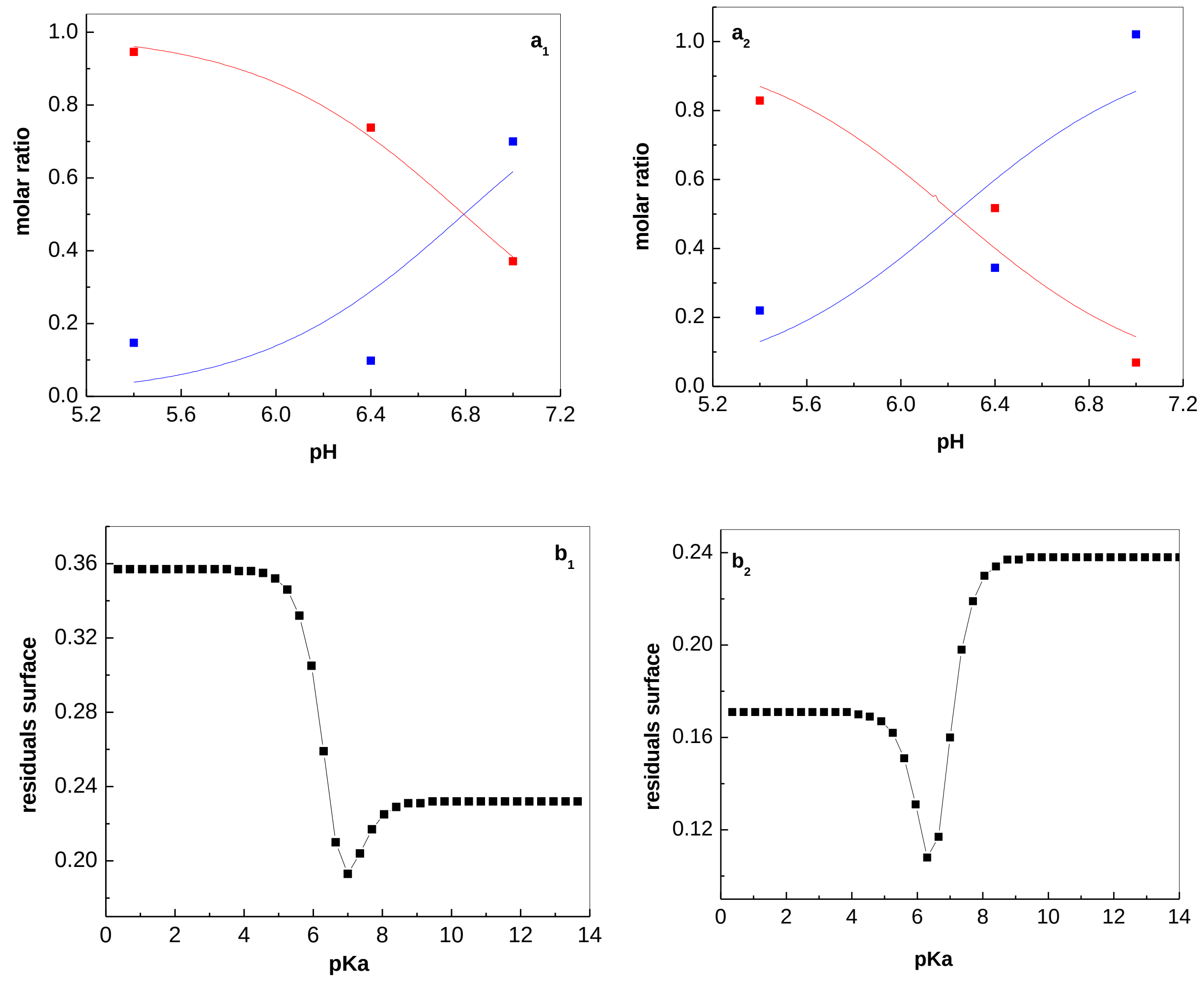

Fig 1S. Concentration distribution diagrams of FA before (a_1) and after UV irradiation (a_2) and their minimization curves (b_1, b_2), respectively.

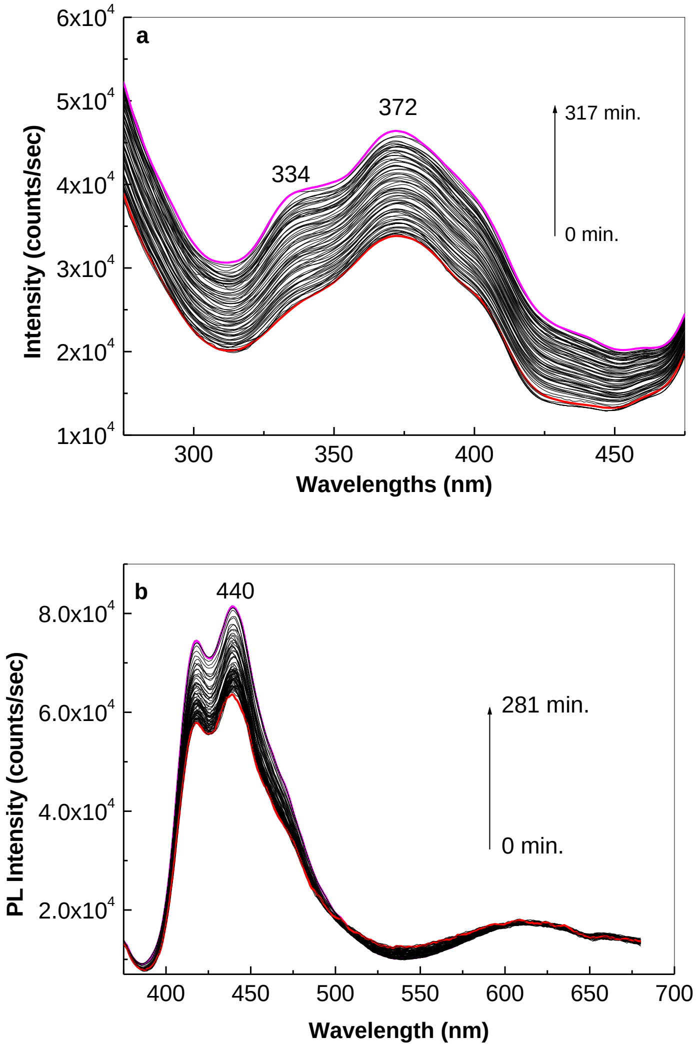

Figure 2S. Photoluminescence excitation (a, the emission wavelength is equal to 500 nm) and photoluminescence (b, excitation wavelength equal to 355 nm) spectra of the folic acid powder under irradiation time of 317 and 280 minutes. The photoluminescence excitation and photoluminescence spectra of the folic acid samples before of irradiation correspond to red curves. Magenta curves correspond to the photoluminescence excitation and photoluminescence spectra of the folic acid samples after an irradiation time equal to 317 and 280 minutes, respectively. Black curves correspond to the intermediate photoluminescence excitation and photoluminescence spectra of the folic acid solution in above phosphate buffers collected each at 141 and 125 seconds, respectively.

Table 1S. Assignment of experimental and calculated IR modes of FA

**Vibrational modes Experimental cm^{-1}**	**Vibrational modes Calculated cm^{-1}**	**Assignments**
696	704	C-H out-of-plane deformation + N-H of NH_2 group in Pt + N-H in C_6H_4 -NH-CH_2
764	766	C-H out-of-plane deformation of alkyl group in GA
839	852	C-H out-of-plane deformation in Pt + C-H bending in GA + N-H bending in amide group + C-C stretching in alkyl group in PAB
912	915	Benzene ring deformation in PAB + C-H bending in GA+ C-N stretching in C_6H_4 -NH-CH_2
974	968	C-H bending in benzene ring
1038-1055	1055	N-H bending in Pt
1107	1103	C-H bending in benzene ring in PAB + alkyl group in GA + O-H bending of COOH group inf GA + N-H bending in amide group
1192	1192	C-H bending of alkyl group in GA
1227	1238	C-H bending in PAB + C-H bending of alkyl group in GA + N-H bending + O-H bending +C-N stretching in Pt
1290	1299	C-H bending + N-H bending + O-H bending in Pt
1338	1337	N-H bending in PAB + C-H bending + N-H bending in Pt + O-H bending + C=O stretching of COOH group in GA
1411	1409	N-H bending + C-H bending in PAB + C-H bending of alkyl group + O-H of COOH group in GA + O-H bending in Pt

1452	1453	C-H bending in PAB+<br>C-H bending of alkyl group in GA +<br>N-H bending of C_6H_5-NH-CO group +<br>N-H bending in NHCO group
1483	1480	Ring deformation + C=N stretching + C-N stretching + O-H bending in Pt
1603	1598	Ring deformation + C=N stretching + C-N stretching in Pt
1689	1682	Ring deformation + C-H bending + COOH group in Pt

Table 2S. Assignment of experimental and calculated IR modes of PABGA

**Vibrational modes Experimental cm^{-1}**	**Vibrational modes Calculated cm^{-1}**	**Assignments**
696	677	C-H out-of-plane deformation in benzene ring
758	745	C-H out-of-plane deformation in benzene ring
842	838	CH out-of-plane in benzene ring + N-H in NH_2 group
887	892	C-H twisting in benzene ring
906	907	N-H in amide group and C-H in alkyl group
948	963	C-C bending in alkyl and benzene ring + C=O stretching in COOH groups
1001-1028	996	C-H in benzene ring + N-H in amine group
1070	1079	N-H scissor in amine group
1115	1100	O-H in COOH + N-H in amide group + C-H in benzene ring
1197	1189	C-H in alkyl group + N-H in amide group + O-H in COOH group
1331	1341	C-H in benzene ring + N-H in amine group
1358	1362	C-H in alkyl group + O-H in COOH group
1450	1457	N-H in amide group + C-H in alkyl group + O-H in COOH groups

1492	1492	C-H in benzene ring + C-H in alkyl group + N-H in amine group + C-N in CH-NH
1713	1733	C=O + N-H in amide group

Table 3S. Assignment of experimental and calculated IR modes of P6CA

Vibrational modes Experimental cm^{-1}	Vibrational modes Calculated cm^{-1}	Assignments
696	696	C-H out-of-plane deformation in Pt ring
758	755	O-H in COOH group
842	839	N-H rocking in NH_2 + C-N in aromatic ring + O-H in COOH
983	978	N-H rocking in NH_2 + O-H group in substituted aromatic ring
1028	1022	N-H in NH_2 and O-H in substituted aromatic ring
1115	1118	O-H in COOH group
1197	1189	O-H in COOH group + O-H in substituted aromatic ring
1450	1469	O-H in COOH group + O-H in substituted aromatic ring + C-N + C=N
1600	1613	C-N in heterocycle and NH in amine group
